# Active Site Architecture and Reaction Mechanism Determination of Cold Adapted β-d-galactosidase from *Arthrobacter* sp. 32cB

**DOI:** 10.3390/ijms20174301

**Published:** 2019-09-03

**Authors:** Maria Rutkiewicz, Anna Bujacz, Marta Wanarska, Anna Wierzbicka-Wos, Hubert Cieslinski

**Affiliations:** 1 Institute of Technical Biochemistry, Faculty of Biotechnology and Food Sciences, Lodz University of Technology, Stefanowskiego 4/10, 90-924 Lodz, Poland; 2 Department of Molecular Biotechnology and Microbiology, Faculty of Chemistry, Gdansk University of Technology, Narutowicza 11/12, 80-233 Gdansk, Poland; 3 Department of Microbiology, Faculty of Biology, University of Szczecin, Felczaka 3c, 71-412 Szczecin, Poland

**Keywords:** galactosidase, hydrolysis, reaction mechanism, complex structures, cold-adapted, GH2

## Abstract

*Arth*βDG is a dimeric, cold-adapted β-d-galactosidase that exhibits high hydrolytic and transglycosylation activity. A series of crystal structures of its wild form, as well as its *Arth*βDG_E441Q mutein complexes with ligands were obtained in order to describe the mode of its action. The *Arth*βDG_E441Q mutein is an inactive form of the enzyme designed to enable observation of enzyme interaction with its substrate. The resulting three-dimensional structures of complexes: *Arth*βDG_E441Q/LACs and *Arth*βDG/IPTG (ligand bound in shallow mode) and structures of complexes *Arth*βDG_E441Q/LACd, *Arth*βDG/ONPG (ligands bound in deep mode), and galactose *Arth*βDG/GAL and their analysis enabled structural characterization of the hydrolysis reaction mechanism. Furthermore, comparative analysis with mesophilic analogs revealed the most striking differences in catalysis mechanisms. The key role in substrate transfer from shallow to deep binding mode involves rotation of the F581 side chain. It is worth noting that the 10-aa loop restricting access to the active site in mesophilic GH2 βDGs, in *Arth*βDG is moved outward. This facilitates access of substrate to active site. Such a permanent exposure of the entrance to the active site may be a key factor for improved turnover rate of the cold adapted enzyme and thus a structural feature related to its cold adaptation.

## 1. Introduction

Glycosyl hydrolases (GHs) are sugar processing enzymes, divided into families based on their structures. This is the reason why the lactose processing β-d-galactosidases (βDGs) belong to different GH families: GH1, GH2, GH35, GH42, and GH59. Their common structural feature is presence of a TIM-barrel type catalytic domain followed by a variety of β-architecture domains, which nature and occurrence differ among GH subfamilies [1].

The most studied β-d-galactosidase in the GH2 family is bacterial *lacZ* βDG from *Escherichia coli* (*Ecol*βDG) [2,3,4]. It is a large homotetramer, where each monomer consists of 1023 amino acids. Its primary mode of action is to catalyze the hydrolysis of lactose to d-galactose and d-glucose. To achieve its full catalytic efficiency *Ecol*βDG requires the presence of divalent ions such as Mg^2+^ or Mn^2+^, which can result in 5−100-fold increase of activation depending on the substrate. Within the *Ecol*βDG catalytic site, two subsites can be distinguished: the first exhibits high specificity for binding the galactose moiety, whereas the second provides a platform for weak binding of different moieties [5]. If an excess of galactose occurs, *Ecol*βDG exhibits transglycosylation activity that results in formation of allolactose, a disaccharide composed of d-galactose and d-glucose moieties linked through a β-(1,6)-glycosidic bond [6,7].

Lactose processing enzymes are commonly used in the dairy industry for production of lactose-free products. Keeping dairy products in refrigerated conditions results in crystallization of lactose that leads to an undesirable grainy texture. That is why lactose removal is used for improving the quality of final products, such as ice-creams and some types of cheeses [8,9].

Another example of an enzyme from GH2 family, commonly used in dairy industry, is yeast βDG from *Klyvuromyces lactis* (*Klyv*βDG) [10]. Similar to *Ecol*βDG, it consists of 1032 aa and its functional form is a homotetramer. Not only it catalyzes lactose hydrolysis, but also transglycosylation reaction that results in formation of galactose derivatives, among other alkyl-galactosides [11], gal-mannitol, [12], and bionic acids [13].

However, the usage of a cold-adapted enzyme, exhibiting similar catalytic efficiency to ones already implemented but at lower temperatures (4–18 °C), is highly sought after. Especially for the food industry, removing the need for a heating step not only brings the costs of production down, but it also prevents potential mesophilic contamination and loss of nutritional value of food products due to heating, and production of unwanted products by thermal conversion [14,15].

*Arth*βDG is an interesting candidate for industrial use. Not only can it hydrolyze lactose at a rate comparable to βDG from *Klyvuromyces lactis* but it exhibits additional transglycosylation activity [16]. Galactooligosaccharides (GOS) and heterooligosaccharides (HOS) are prebiotics, which are important for human health. That is why, with constantly increasing evidence of their consumption benefits, they found their way into infant nutrition and special nutrition, and more recently have become increasingly present in everyday food products [17,18,19,20,21,22,23,24,25,26].

The modification of transglycosylase activity specificity and efficiency may be achieved by controlling reaction equilibrium or by enzyme engineering. Studies concentrated on introducing mutations into subsites of GHs showed that the modulation of hydrolysis and transglycosylation activities can be achieved by means of knowledge-based enzyme engineering. However, the role of individual amino acids in the active site must be discovered as a basis for successful design of an enzyme with specific, desired activities [27].

*Arth*βDG is a five-domain protein of molecular weight 110 kDa. The catalytic domain, in form of TIM barrel, is surrounded by three IG-like domains and, as typical for the GH2 family, an N-terminal super β-sandwich domain. Regardless of low sequence identity, this monomer’s architecture is strikingly similar to *Klyv*βDG and *Ecol*βDG, which enabled determination of catalytic residues E441 and E517. Cold-adapted *Arth*βDG possesses a functional dimer (not typical for the GH2 family). The dimer is stabilized by head-to-tail interactions between Domains 1 and Domains 5 from neighboring molecules [28]. The same oligomerization state, though shaped differently, was previously described by us for another cold-adapted β-d-galactosidase from *Paracoccus* sp. 32d for which we had determined crystal structure [29]. Thanks to comparative analysis, the structural features that may play a key part in its cold-adaptation were described. Most interesting was maximization of energy gain from the surface residue–solvent interactions, that was obtained by reduction of oligomerization state and formation of hydrophobic patches on the protein’s surface [30].

The comprehensive structural study of cold-adapted *Arth*βDG reaction mechanism is a first necessary stage for the knowledge-based enzyme engineering that could lead to creation of an enzyme that would not only hydrolyze lactose, but also effectively convert it to the beneficial for human health GOS and HOS at cold conditions. Usage of native protein would limit us to analysis of substrate binding using substrate analogs which could not by hydrolyzed by the enzyme, such as isopropyl β-d-1-thiogalactopyranoside (IPTG), or less preferable substrates such as ortho-nitrophenyl-β-galactoside (ONPG). However, thanks to designing an inactive mutant in which catalytic E441, acting as acid catalyst, was substituted with the structurally isomorphous glutamate residue, we were able to obtain complexes with the natural substrate lactose bound in both deep and shallow binding modes.

## 2. Results

### 2.1. Crystal Structures of ArthβDG and ArthβDG_E441Q Complexes

**Figure 1:**
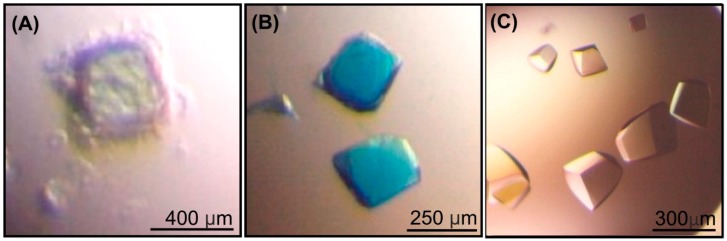
The crystals of *Arth*βDG: after addition of ONPG (**A**); after 2 h of soaking in X-gal (**B**). The crystals of *Arth*βDG_E441Q mutein soaked 24 h with mixture of lactose and galactose (**C**).

During soaking of *Arth*βDG crystals with ONPG and X-gal (5-bromo-4-chloro-3-indolyl-β-D-galactopyranoside) a coloration of crystals was observed. It was rapid for crystals soaked with X-gal, as they turned intensely blue within 3 min (Figure 1B).

Different short soaking times (in a range of 10 s to 10 min, 22 crystals tested) and excess of X-gal (from 3 to 20 molar access in respect to protein concentration) gave intensely blue coloration of soaked crystals. This indicates that the enzyme in the crystal was in its active form and performed hydrolysis of X-gal. The structure solved using diffraction data collected from blue crystals was identical with the *Arth*βDG/GAL obtained by soaking with lactose. It means that X-gal, similarly to lactose, was hydrolyzed by enzyme during soaking of native crystals in ligand solution. Blue color came from 5,5′-dibromo-4,4′-dichloro-indigo—a dimer of the second product (5-bromo-4-chloro-3-hydroxyindol) of X-gal hydrolysis. The blue dye is either deposited in solvent channels of the crystal or randomly bound to the surface of protein. If the dimerized product of X-gal hydrolysis would be bound specifically, it should be detectible in the resulting crystal structure, because it contains two Br atoms, which give strong picks on the electron density maps.

Soaking of *Arth*βDG crystals with ONPG resulted in a yellow halo around the soaked crystals (Figure 1A); however, the rate of ONPG hydrolysis was probably lower than X-gal as the coloration was visually observed only after more than an hour soaking. For the crystal soaked in ONPG for 15 min, the color was not observed and the complex of *Arth*βDG with ONPG bound was formed - an intact ONPG was visible in the active site after determining the crystal structure.

Crystals of *Arth*βDG soaked with lactose underwent fast deterioration, and a very short soaking time was required to obtain crystals still suitable for diffraction experiments. The diffraction data collected after only 1 min of soak with lactose resulted in the crystal structure with galactose. This is evidence that the active enzyme performed hydrolysis of lactose in crystal, in such fast rate, and product of reaction–galactose, bound in active site was visible in electron density maps.

The soaking of *Arth*βDG_E441Q mutein crystals with lactose yielded no complex in solved crystal structures, as for soaking times up to 6 h no ligand was found in the structure and for longer soaking times crystals were destroyed. We have succeeded in determining the crystal structure of *Arth*βDG_E441Q complex with lactose after soaking crystals of *Arth*βDG_E441Q with mixtures of lactose-galactose (Figure 1C) and lactose-fructose for 24 h. After this time, crystals of the mutein complexes were still without visible signs of deterioration and resulted in very good resolution data. The complex structures of lactose bound in shallow and deep mode were obtained while subjecting those crystals to diffraction experiments.

The crystal structures of *Arth*βDG complexes with galactose (2.1 Å), IPTG (2.2 Å), ONPG (2.8 Å), and its mutein *Arth*βDG_E441Q (1.8 Å) in complexes with lactose (LAC) bound in shallow mode *Arth*βDG_E441Q/LACs (1.9 Å) and in deep mode *Arth*βDG_E441Q/LACd (1.8 Å) were processed in trigonal space group *P3_1_21*, the same as *Arth*βDG native structure. Matthew’s volume calculation [31] revealed that no changes in crystal packing were detected, and protein monomer was present in each asymmetric unit. Crystal structures of these complexes were solved in PHENIX by isomorphous replacement using rigid body procedure and the native structure of *Arth*βDG (PDB ID: 6ETZ) as a model. This allowed us to obtain the molecule in the same position and orientation in all the analyzed structures, which facilitated comparison of not only the structures, but also electron densities. The details for the diffraction data collection and processing are presented in Table 1. Each structure was further refined in PHENIX.REFINE, including TLS parameters [32] defined for each domain. The resulting refinement statistics are given in Table 1.

### 2.2. Active Center of ArthβDG and ArthβDG_E441Q Mutein

**Figure 2:**
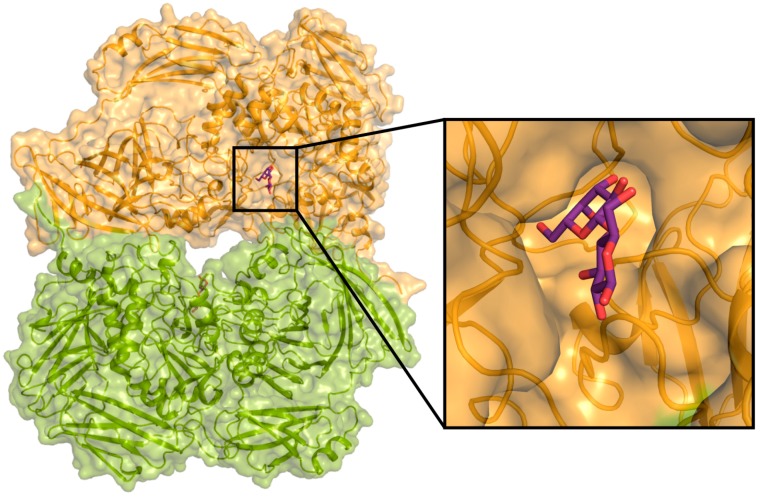
The dimer of *Arth*βDG_E441Q and the zoom of one of the active site cavities with lactose.

**Figure 3:**
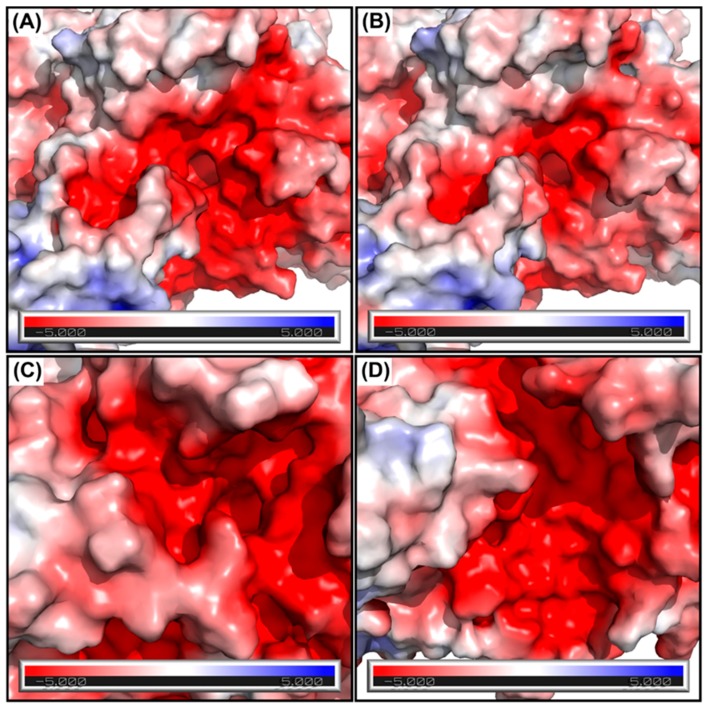
The surface potential visualization at the active site of *Arth*βDG (**A**), *Arth*βDG_E441Q (**B**), *Ecol*βDG (**C**), and *Klyv*βDG (**D**).

Catalytic site of *Arth*βDG, located at the bottom of a relatively wide funnel on the top of catalytic Domain 3, is complemented with parts of the chain from Domain 1 and Domain 5 at the entrance of the active cavity (Figure 2). The funnel leading to the active site has a strongly acidic character, which is beneficial for binding of carbohydrate substrates (Figure 3).

The active site cavity has an acidic character throughout, which facilitates the binding of the saccharide substrate, which is typically lactose. Such a shape of the active site cavity is observed for other βDGs with transglycosylation activities: *Ecol*βDG and *Klyv*βDG [4,7,10] as it facilitates the binding of a larger acceptor of galactosyl group, such as galactose, fructose, or salicin. *Arth*βDG forms a widely open entrance to its catalytic site, which makes it more accessible for the substrate but also promotes product dissociation. Both, easier product dissociation and active sites not being shielded or restricted from solvent may be considered as structural cold-adaptation as it influences the enzymes’ turnover rate.

### 2.3. Structural Analysis of Reactions’ Mechanism Catalyzed by ArthβDG

**Figure 4:**
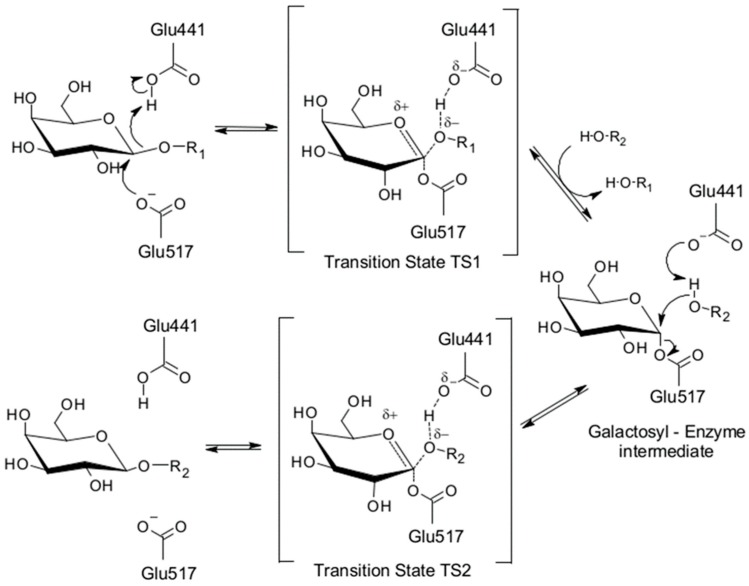
The reaction mechanism of Koshland double displacement with the catalytic residues numbered as for *Arth*βDG [33].

All determined crystal structures showed precisely the changes in the active site of the enzyme in different stages of catalyzed reaction. Visualizing these structural changes helps to understand and explain a classical Koshland double-displacement mechanism occurring during hydrolysis of (1,4)-β-O-glycosidic bond catalyzed by *Arth*βDG (Figure 4).

Determined crystal structures of *Arth*βDG complexes with specific ligands may be divided into three groups: complexes with substrates and their analogues (early complexes) *Arth*βDG_E441Q/LACs, *Arth*βDG/IPTG; the second group are intermediates complexes *Arth*βDG_E441Q/LACd *Arth*βDG/ONPG; and the third one is the complex with product *Arth*βDG/GAL.

The early complexes with substrate show LAC and IPTG binding in shallow mode, intermediate complexes depicts deep binding of substrate that directly precedes formation of galactosyl-enzyme covalent bond, and the product complex allows description of the product release process.

**Figure 5:**
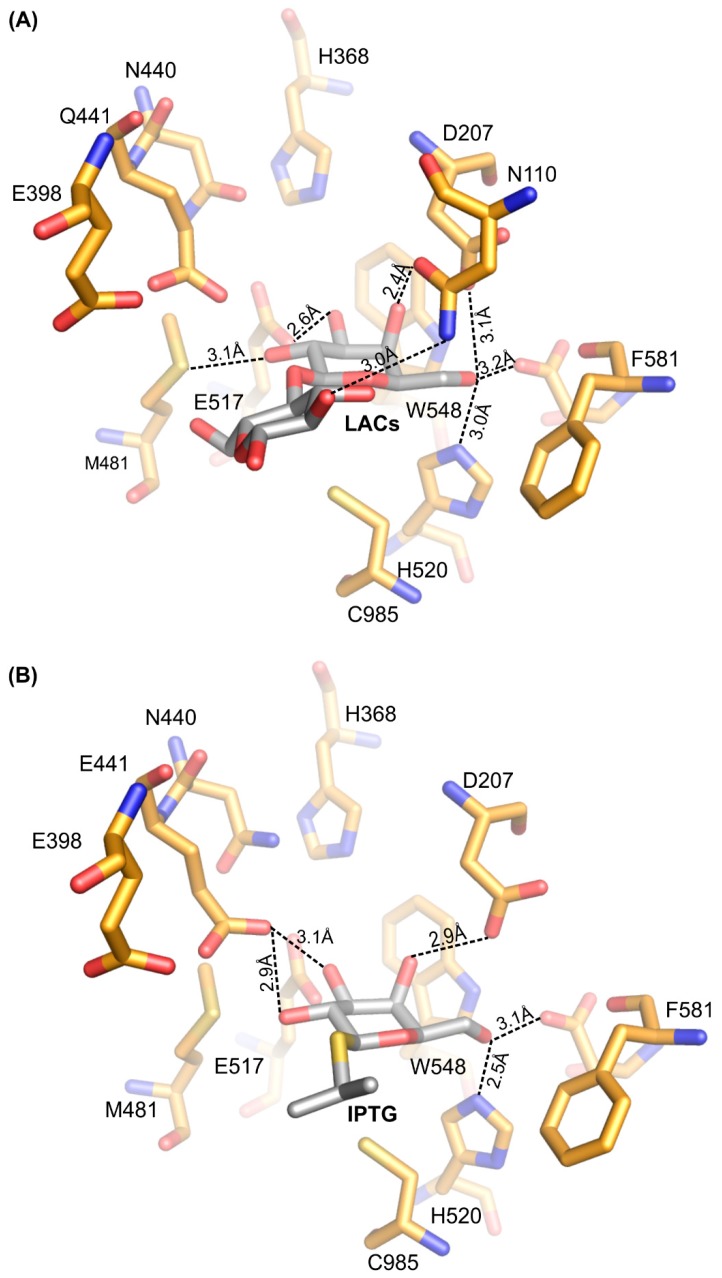
Early complexes of *Arth*βDG with saccharide substrate and substrate analogue: the molecule of lactose (**A**) and IPTG (**B**) bound at shallow binding site.

At the early stage of the reaction, the substrate is bound in the shallow binding site where the galactosyl group is stabilized by a number of H-bonds between its hydroxyl groups and residues N110, E441, E517, M481, H520, and H368 via water molecules and by an interaction with a sodium ion. Additionally, the glucosyl moiety of lactose is stabilized by H395 and E398 via a water molecule, even though there is already an interaction between substrate and catalytic residues (E441 and E517), and the position of the substrate does not allow access to O-glycosidic bond (Figure 5).

**Figure 6:**
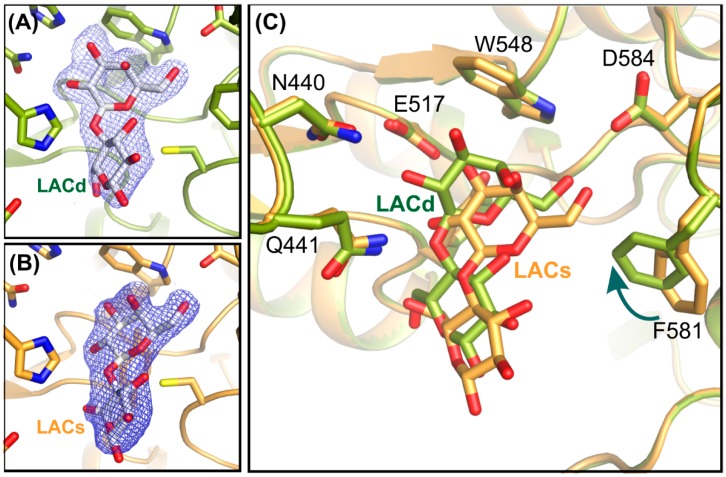
Enzyme active site of shallow and deep binding of lactose. Electron density 2F_o_-F_c_ map of lactose in deep (**A**) and shallow (**B**) binding mode (contoured at 1σ). Superposition of enzyme active site in both structures (**C**).

The insertion of substrate into deep binding is associated with movement of F581 phenyl ring, which rotates around Cα-Cβ bond causing shift of aromatic ring by 2.9 Å in the direction of the active center, reducing the volume of the shallow binding site (Figure 6). Surprisingly, no movement of backbone is observed during the transfer of ligand from shallow to deep binding site. During substrate transfer into the deep binding site, the galactosyl moiety, properly positioned in shallow binding stage, is moved deeper into the active site by approximately 2.4 Å being at the same time rotated around an axis perpendicular to the sugar ring by approximately 60°.

**Figure 7:**
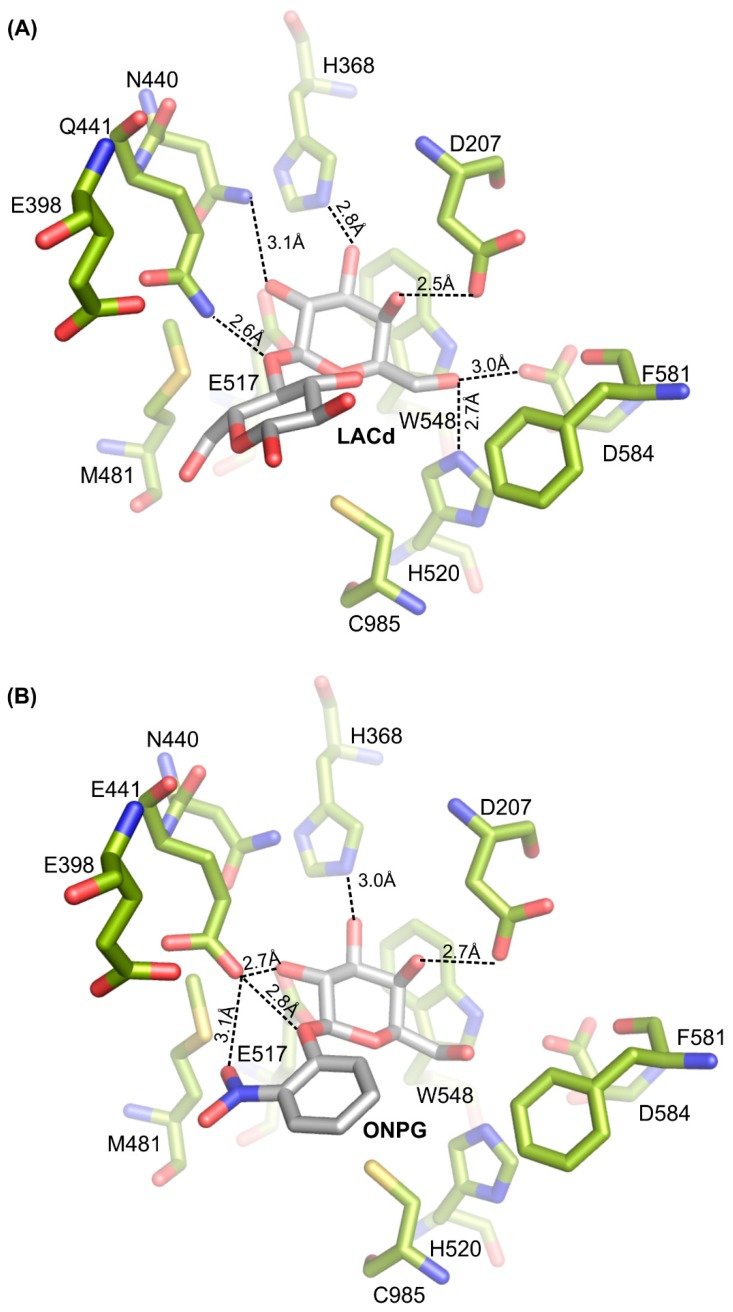
Late complexes of *Arth*βDG with substrates: the molecules of lactose (**A**) and ONPG (**B**).

In the deep binding site of *Arth*βDG, the galactosyl ring is stabilized by direct interactions of its hydroxyl groups with E441 (Q441 in mutein), E517, H368, D207, sodium ion and N440, D584, H520 (latter observed by deeper bound lactose in complex of *Arth*βDG_E441Q_LACd). Now in *Arth*βDG_E441Q_LACd, the NH_2_ from the amide group of Q441 interacts directly with oxygen from the glycosidic bond of lactose, which is 2.6Å away (Figure 7A). In *Arth*βDG/ONPG, the carboxyl group of E441 interacts directly with oxygen from the glycosidic bond of ONPG, which is at a distance of 2.8 Å (Figure 7B).

**Figure 8:**
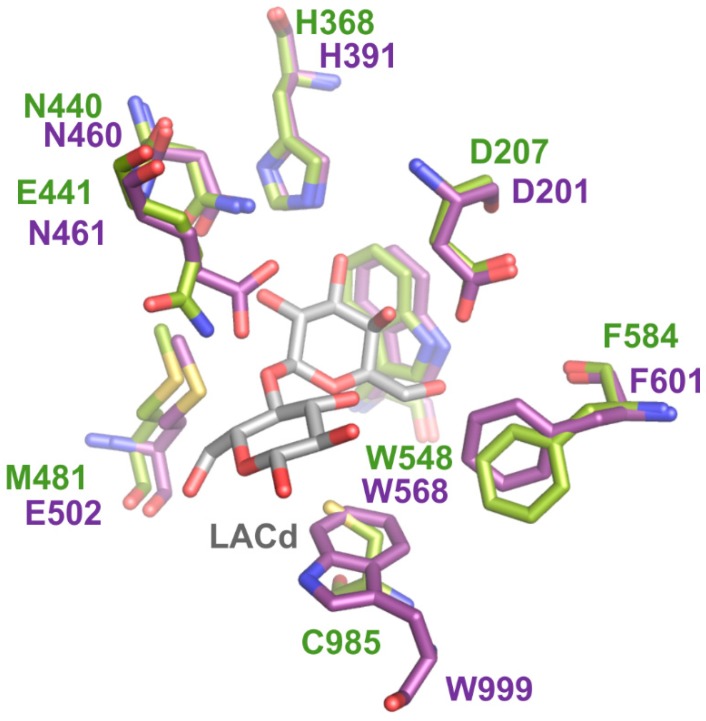
Superposition of catalytic sites of *Arth*βDG with lactose bound in deep mode (green) and *Ecol*βDG (purple).

The superposition of active sites of *Arth*βDG with *Ecol*βDG shows the conservation of amino acids involved in stabilization of the galactosyl moiety. However, these two enzymes differ in the stabilization of a second moiety of β-d-galactoside (Figure 8). If we consider binding of the natural substrate, lactose, the second moiety is glucopyranose. In the active site of *Ecol*βDG, the glucopyranose ring is stabilized by π-stacking interaction with W999. However, in the *Arth*βDG active site, W999 is substituted by C985. This cysteine residue does not influence stabilization of substrate in shallow binding mode. However, when lactose is bound in deep mode, the center of the glucopyranose ring is at a distance of 4.4 Å from C985 making π-sulphur interaction possible. Thus, substitution of W999 with C985 reduces stabilization of the second moiety of β-d-galactoside during shallow binding mode, but is still creating stabilizing interactions when the substrate is bound in deep mode. Furthermore, this results in creating more space in the close vicinity of the active site by substituting the bulky indol group with a smaller cysteine residue side chain.

**Figure 9:**
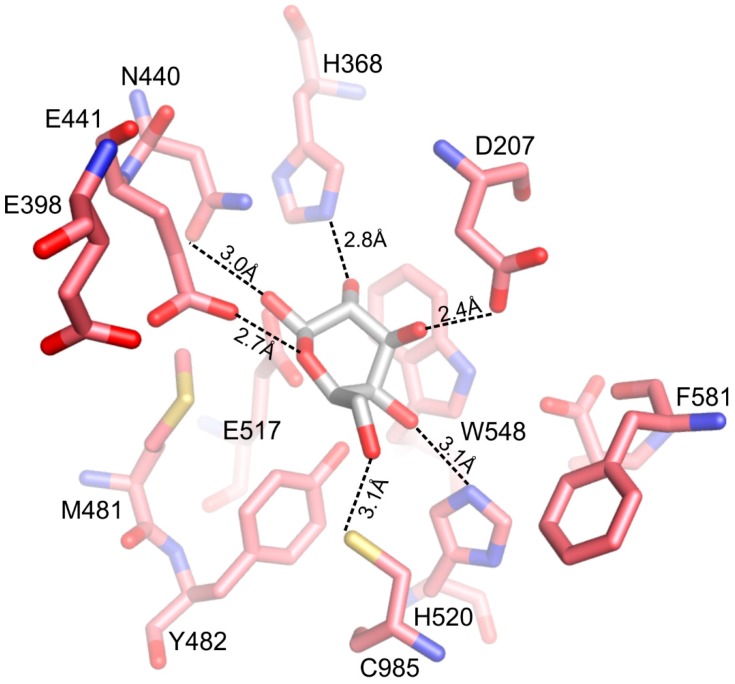
Complex structure of *Arth*βDG with galactose in half-chair conformation bound in the active center.

After the hydrolysis reaction is completed, the F581 side chain moves back into its previous position, opening the way for galactose molecule evacuation from the active site (Figure 9).

The product, now in half-chair conformation, is still stabilized by a number of interactions: D207, H368, N440, E441, Y482, E517, H520, and C985 (Figure 7). The ‘open’ position of F581 is also observed for unliganded structures of *Arth*βDG (PDB IDs: 6ETZ) and its mutant *Arth*βDG_E441Q, suggesting that its movement is dependent upon substrate presence at the shallow binding site.

## 3. Discussion

GH2 family β-d-galactosidases are sugar configuration-retaining enzymes that follow a classical Koshland double-displacement mechanism. These crystal structures of *Arth*βDG complexes with ligands enabled characterization of the active site and determined which residues take part in two modes of substrate binding: deep and shallow.

The large rotation of the galactosyl residue during deep binding would most probably result in forming π-stacking interaction with W548. Such a form of intermediate stabilization was described for *Ecolβ*DG [7]. It should be noted that a tryptophan residue is the preferred aromatic amino acid for binding of carbohydrates [34,35] and is frequently present in carbohydrate binding domains of proteins. In the case of *Arth*βDG, only one tryptophan, W548, is located in the bottom of the active site. Additionally, three tryptophan residues are present at the entrance to the catalytic pocket (W402, W470, and W773), where they may form platforms for initial sugar binding. Another amino acid which is considered to play an important role in carbohydrate binding is histidine. The active site of *Arth*βDG contains several histidine residues: H334, H368, H395, H520, and H553. Among them, H368 and H520 are directly involved in stabilizing the galactosyl moiety. H520 is primarily involved in stabilization of hydroxyl group O6 of the galactosyl moiety during shallow binding of substrate. When the substrate is moved deeper into catalytic site, H368 stabilizes the position of hydroxyl group O3. It must be noted that the catalytic site architecture of *Arth*βDG is composed such a way that only a sugar moiety with a proper conformation of hydroxyls O2, O3, and O4 can be effectively bound in the active site. Hence, residues forming H-bonds with hydroxyl groups in these positions H368, N440, and D207, play a crucial role in enzyme’s specificity.

It is worth noting that a typical chair conformation of the galactosyl ring in substrate (^1^*C*_4_) is changing to a half-chair (^3^*H*_4_)^hkkkkHhhh^ conformation in the still bound product of the half-reaction. There are many conformations of pyranose ring possible in solution; however, some of them are more stable than others. In the case of lactose, it usually has a relaxed chair conformation in solution. The double displacement mechanism, in which lactose is hydrolyzed by retaining galactosidases, such as *Arth*βDG, undergoes formation of two oxocarbenium ion-like transition states (Figure 4). Such transition states must be formed with sp^2^ hybridization and formation of a positive charge on anomeric carbon atom of the substrate. Only a few conformations of galactosyl moiety allow sp^2^ hybridization on anomeric carbon, one of which is half-chair conformation ^3^*H*_4_, observed for the galactose bound in active site of *Arth*βDG [36].

The rotation of F581 (F601 in *Ecol*βDG) was described as one of the factors associated with the deep binding mode, together with 10 Å movement of a 10-aa loop from Domain 5. However, in the case of *Arth*βDG, the movement of F581 and D207 are the only conformational changes accompanying the reaction mechanism. In fact, the 10-aa loop in *Arth*βDG is stabilized by a number of strong interactions with other parts of Domain 5, in a position allowing better access of the substrate to the active site. It should be noted that it is one of the regions of *Arth*βDG in which the backbone differed significantly from homologous structures. These facts lead us to consider this permanent exposure of the entrance to the active site as a structural adaptation towards activity in cold conditions. Fewer structural hindrances for substrate entering and product leaving the active site can result in a higher turnover rate. Analysis of these obtained crystal structures shows that *Arth*βGD forms a widely open entrance to its catalytic site, which makes it more accessible for the saccharide substrate and promotes product dissociation.

Both galactosyl binding sites, shallow and deep ones, form a net of H-bonds that stabilize this part of the substrate. On the other hand, the glucosyl moiety of lactose, or the non-galactose moieties of IPTG and ONPG, are hardly stabilized by any interactions during shallow binding. The *Ecol*βDG W999 is substituted at *Arth*βDG with a cysteine residue which may stabilize the second sugar ring of the substrate by π-sulphur interactions during deep binding, however such a substitution would render the enzyme less specific toward binding a sugar moiety at this position. Thus, not only disaccharides, but also other galactosides are processed by *Arth*βDG. The enzyme’s lack of preference for the second moiety in galactoside may be the main reason for its ability to hydrolyze a wide variety of substrates, as well as for its ability to transfer galactosyl group to a variety of acceptors [16] resulting in an interesting range of potentially useful heterooligosaccharides.

## 4. Materials and Method

### 4.1. Site-Directed Mutagenesis of Gene Encoding ArthβDG

The gene encoding the *Arth*βDG enzyme, which was previously cloned into the pBAD/Myc-His A expression vector [16], has been mutated in a site-specific manner using the Q5 Site-Directed Mutagenesis Kit (NEB, Ipswich, MA, USA) following the manufacturer’s protocol. For this purpose, a pair of mutagenic primers was designed and synthesized (Genomed, Warszawa, Poland). Primer ForBglAr32cBm441: 5′GTCCCTGGGCAACCAGGCGGCACCGG3′ and primer RevBglAr32m441: 5′CACATGACCACCGAGGCGTGGTTCTTGTCGCGC3′ allowed us to introduce a point mutation at 1321 nucleotide position in the gene substituting G with C resulting in the substitution of glutamic acid (E) residue with glutamate (Q) residue in the 441 position of the amino acid chain of *Arth*βDG. Hence, the product of mutated gene expression has been called *Arth*βDG_E441Q. In theory, this amino acid change should abolish β-d-galactosidase activity of mutein *Arth*βDG_E441Q.

PCR cycling conditions were as follows: (1) Initial DNA denaturation at 98 °C for 30 s; then (2) 25 cycles of PCR product amplification consisting of 10 s of DNA denaturation at 98 °C, 20 s of mutagenic primers annealing at 70 °C, and 3 min 20 s of PCR product extension at 72 °C; and (3) the final PCR product extension at 72 °C for 7 min. After PCR, the amplified DNA product was directly added to unique Kinase-Ligase-DpnI (KLD) enzymes mix. Then the product of KLD reaction (5 min at room temperature) was directly used to transform NEB 5-alpha chemically competent *E.coli* cells (the *lacZ* deletion mutant, ∆ (lacZ) M15). After that, transformants were spread on Luria–Bertani agar plates (10 g L^−1^ of peptone K, 5 g L^−1^ of yeast extract, 10 g L^−1^ of NaCl, and 15 g L^−1^ of agar) supplemented with ampicillin (100 μg mL^−1^), X-Gal (40 μg mL^−1^) and l-arabinose (200 μg mL^−1^). After plate incubation—firstly at 37 °C for 12 h, and then at 22 °C for next 12 h—a few recombinant colonies without β-d-galactosidase activity were chosen for further studies. Plasmids isolated using the ExtractMe Plasmid DNA Kit (Blirt, Gdansk, Poland) from selected recombinants were sequenced (Genomed, Warszawa, Poland) and analyzed (blast2go on-line tool). Recombinant plasmid pBAD-Bgal32cB_E441Q(A) harboring the properly mutated *Arthrobacter* sp. 32cB β-d-galactosidase gene under the control of the P_BAD_ promoter was used for effective production of *Arth*βDG_E441Q mutein in *E. coli* host [16].

### 4.2. Expression and Purification of ArthβDG and ArthβDG_E441Q

Heterologous expressions of recombinant *Arth*βDG and *Arth*βDG_E441Q proteins were performed in the *E. coli* LMG 194 cells transformed with pBAD-Bgal32cB and pBAD-Bgal32cB_E441Q plasmids, respectively, as previously described. [25] Both proteins were purified by two ion-exchange chromatography steps (weak anion exchanger and strong anion exchanger), followed by a size-exclusion chromatography step.

The fractions containing *Arth*βDG were identified by SDS-PAGE electrophoresis run on 10% SDS-polyacrylamide gel and by enzymatic activity assay with ONPG as a substrate [25], whereas the fractions containing *Arth*βDG_E441Q were identified by SDS-PAGE only, due to lack of enzymatic activity. The sample buffer was changed into 0.05 M HEPES pH 7.0 and the samples were concentrated using 50 kDa cut-off membrane Vivaspin filters (Sartorius, Goettingen, Germany) up to the protein concentration of 15 mg/mL.

### 4.3. ArthβDG Crystallization and Diffraction Data Collection

Crystals of *Arth*βDG and *Arth*βDG_E441Q mutein were grown using the same optimization matrix of 25–45% Tacsimate^TM^ and pH ranges between 6.0–8.0. All the drops were set up using a seed stock prepared from crystals of *Arth*βDG grown at 35% Tacsimate^TM^ pH 7.0 and diluted 10,000 times. Numerous attempts of co-crystallization with ligands were undertaken but no crystal structures of desired complexes were obtained. Furthermore, addition of natural substrate, lactose, prevented formation of *Arth*βDG_E441Q crystals even at very low concentration of added ligand. Crystal structures of investigated *Arth*βDG and *Arth*βDG_E441Q complexes were obtained by soaking of native and mutant crystals with desired ligand or ligands mixture. Soaking was performed by adding powder of ligand directly to the crystallization drop. The soaking experiments were performed for 15 min, 30 min, 1 h, 2 h, 6 h, 14 h, and 24 h prior to flash-freezing. The crystals, prior to mounting and flash-freezing, were protected with 60% Tacsimate^TM^ of pH corresponding to crystallization conditions [37].

High-resolution diffraction data were collected using synchrotron sources on beamlines 14.1 and 14.2 at BESSY, Berlin, Germany and P13 beamline at PETRA, DESY Hamburg, Germany. The diffraction images were collected with fine slicing 0.1° and diffraction data were processed using XDSapp [38]. Crystal structures were solved and refined using the PHENIX program suite [39]. As a model, the structure of *Arth*βDG (PDB ID: 6ETZ) was used.

## Figures and Tables

**Table 1:** Diffraction data collection, processing, and refinement statistics for crystal structures of investigated *Arth*βDG complexes.

	*Arth*βDG_E441Q PDB ID: 6SE8	*Arth*βDG_LACs PDB ID: 6SE9	*Arth*βDG_LACd PDB ID: 6SEA	*Arth*βDG_IPTG PDB ID: 6SEB	*Arth*βDG_ONPG PDB ID: 6SEC	*Arth*βDG_GAL PDB ID: 6SED
Diffraction source	P13 PETRA, Hamburg, Germany	BL 14.1 BESSY, Berlin, Germany	BL 14.1 BESSY, Berlin, Germany	BL 14.2 BESSY, Berlin, Germany	BL 14.2 BESSY, Berlin, Germany	BL 14.2 BESSY, Berlin, Germany
Wavelength (Å)	0.976250	0.918400	0.918400	0.918400	0.918400	0.918400
Temperature (K)	100 K	100 K	100 K	100 K	100 K	100 K
Detector	PILATUS 6M	PILATUS 3S 2M	PILATUS 3S 2M	PILATUS 3S 2M	PILATUS 3S 2M	PILATUS 3S 2M
Rotation range per image (°)	0.05	0.1	0.1	0.2	0.2	0.2
Total rotation range (°)	160	180	180	180	180	180
Exposure time per image (s)	0.1	0.2	0.2	0.3	0.3	0.3
Space group	P3121	P3121	P3121	P3121	P3121	P3121
*a*, *b*, *c* (Å)	136.8, 136.8, 127.0	138.9, 138.9, 127.9	138.6, 138.6, 127.4	137.1, 137.1, 126.9	137.4, 137.4, 126.8	136.8, 136.8, 126.8
α, β, γ (°)	90, 90, 120	90, 90, 120	90, 90, 120	90, 90, 120	90, 90, 120	90, 90, 120
Mosaicity (°)	0.133	0.084	0.72	0.115	0.247	0.130
Resolution range (Å)	50.0–1.8 (1.9–1.8)	47.0–2.0 (2.1–2.0)	46.9–1.9 (2.0–1.9)	46.6–2.2 (2.3–2.2)	46.6–2.6 ( 2.7–2.6)	50.0–2.1 (2.2–2.1)
No. of unique reflections	118,383	95,079	109,583	75,085	43,328	80,260
Completeness (%)	98.8 (92.5)	99.4 (97.0)	99.9 (99.5)	99.9 (99.7)	99.4 (98.1)	99.8 (99.3)
Redundancy	7.75 (6.33)	10.09 (10.23)	9.68 (9.85)	5.60 (5.58)	6.63 (6.32)	10.08 (9.66)
I/σ (I)	13.94 (2.12)	15.23 (1.17)	13.86 (1.12)	11.86 (1.01)	11.75 (1.03)	16.07 (1.72)
*R*_meas_ (%)	8.6 (64.5)	10.8 (193.7)	9.8 (177.8)	14.2 (170.6)	16.8 (165.0)	13.6 (140.6)
Overall B factor: Wilson plot/refinement (Å2)	37.8/30.3	47.3/38.8	43.7/36.1	47.2/41.7	60.2/58.3	43.1/37.7
No. of reflections: working/test set	118,348/2101	100,463/2091	116,171/2088	63,550/2101	67,872/2112	66,731/2101
*R*/*R*_free_	0.135/0.165	0.203/0.238	0.182/0.205	0.159/0.205	0.174/0.240	0.166/0.204
No. of non-H atoms: Protein/Ligand/Water	7794/140/826	7619/133/400	7652/97/671	7649/64/617	7624/30/135	7672/65/605
R.m.s. deviations: Bonds (Å)/Angles (°)	0.008/0.964	0.003/0.602	0.010/1.004	0.007/0.873	0.008/1.008	0.002/0.563
Ramachandran plot: Most favored/allowed (%)	97.4/2.6	96.8/3.2	97.6/2.4	97.1/2.9	94.9/5.1	97.2/2.8

## Abbreviations

*Arth*βDG	β-d-galactosidase from *Arthrobacter* sp. 32cB
*Ecol*βDG	β-d-galactosidase from *Escherichia coli*
*Klyv*βDG	β-d-galactosidase from *Klyvuromyces lactis*
GAL	galactose
GOS	galactooligosaccharides
HOS	heterooligosaccharides
IPTG	isopropyl β-d-1-thiogalactopyranoside
Lac	Lactose
ONPG	ortho-nitrophenyl-β-galactoside
X-gal	5-bromo-4-chloro-3-indolyl-β-D-galactopyranoside
